# Effectiveness of Rehabilitation Intervention on Overactive Bladder After Spinal Aneurysmal Bone Cyst Surgery in A Child: A Case Report

**DOI:** 10.7759/cureus.76453

**Published:** 2024-12-27

**Authors:** Hoang Khanh Chi, Kieu Ngoc Quy, Nguyen Quang Anh, Pham Van Minh

**Affiliations:** 1 Rehabilitation, Hanoi Rehabilitation Hospital, Hanoi, VNM; 2 Rehabilitation, Hanoi Medical University, Hanoi, VNM

**Keywords:** clean intermittent catheterization, neurogenic bladder, overactive detrusor, urodynamic, urotherapy

## Abstract

Managing overactive bladder (OAB) in children is recommended to involve rehabilitation intervention including urotherapy, clean intermittent catheterization (CIC), and medication. However, there is scarce evidence on the management of OAB in children in Vietnam, as well as the effectiveness of combining urotherapy, CIC, and medication in managing this condition. We report a case of an 11-year-old female pediatric patient with OAB following aneurysmal bone cyst (ABC) surgery. She was diagnosed with a T8 vertebral ABC at the age of seven years and underwent curative surgery with spinal fusion. Postoperatively, she was diagnosed with neurogenic bladder and was prescribed anticholinergic medication along with CIC. However, her caregivers reported that the treatment did not alleviate her symptoms, and they soon discontinued the regimen due to a limited understanding of her conditions, lack of guidance on CIC techniques, and difficulties in positioning or managing the patient’s pain during catheterization. She did not practice urotherapy exercises, such as pelvic floor or bladder training. Examination in this admission revealed OAB symptoms, including urinary urgency, frequency, nocturia, detrusor overactivity, reduced bladder capacity, and reduced sensation of bladder filling. She was provided with standard and specific urotherapies. Standard therapy included education about her condition, advice on adequate fluid intake, a bladder diary, and recommendations to record fluid intake and output. Specific therapy included bladder training, pelvic floor muscle exercises, double voiding, deferment exercises, and timed voiding, along with 5 mg/day of oxybutynin. After six weeks of treatment, her OAB symptoms improved significantly, with no further detrusor overactivity, increased bladder capacity, and an overall improvement in quality of life. The combination of urological therapy, CIC, and anticholinergic medication can improve clinical symptoms and urodynamic parameters of OAB in children.

## Introduction

Spina bifida is the most frequent cause of neurogenic bladder in children, followed by other congenital anomalies or acquired conditions [1]. Neurogenic bladder has been observed in 3/17 cases of aneurysmal bone cyst (ABC) in the vertebrae [2]. The combination of clean intermittent catheterization (CIC) and medication has been the standard treatment for managing overactive bladder (OAB). Recently, according to the clinical recommendations of the International Children’s Continence Society (ICCS), urotherapy is considered the first-line treatment for most types of lower urinary tract disorders, including neurogenic bladder. Urotherapy, including standard and specific regimens, yields the best outcomes across various urinary disorders [3]. Recently updated guidelines have also recommended combining urotherapy, CIC, and medication as a comprehensive treatment [4,5].

In Vietnam, managing neurogenic bladder with CIC and medication has been implemented for children since 2010, but only at a handful of medical facilities, primarily for children with spina bifida. According to a report in 2023, CIC was shown to reduce urinary incontinence, increase bladder capacity, and lower the incidence of vesicoureteral reflux in children after spinal cord and meningeal surgery [6]. Meanwhile, ABCs originating in the spine are quite rare, and there are almost no reports on the outcomes of neurogenic bladder treatment in children with this condition nor on the effectiveness of combining CIC, medication, and urotherapy for managing neurogenic bladder in these patients.

In this report, we present a clinical case of an 11-year-old girl who experienced bladder dysfunction for four years after a spinal ABC compressing the spinal cord at the age of seven years. The child underwent tumor resection, curettage, and bone grafting immediately upon diagnosis. Neurogenic bladder management was initiated after the surgery, and she received a combination of medication and CIC. However, the treatment was unsuccessful due to poor compliance with CIC by the child and her family. Consequently, her urinary dysfunction persisted for four years, with symptoms of urinary incontinence, urgency, and nocturia. She wore diapers during school hours and recently faced self-esteem issues due to her condition, influencing her quality of life. Upon admission to our department, the importance of supporting her and her family in sustaining long-term treatment appears apparent. In addition to conventional therapies, concurrent cognitive-behavioral therapy is also necessary. Following the ICCS guidelines, we implemented a combination of urotherapy, CIC, and anticholinergic medication. The results showed remarkable improvements in her urodynamic parameters, Overactive Bladder Symptom Score (OAB-SS), and overall quality of life.

## Case presentation

**Figure 1:**
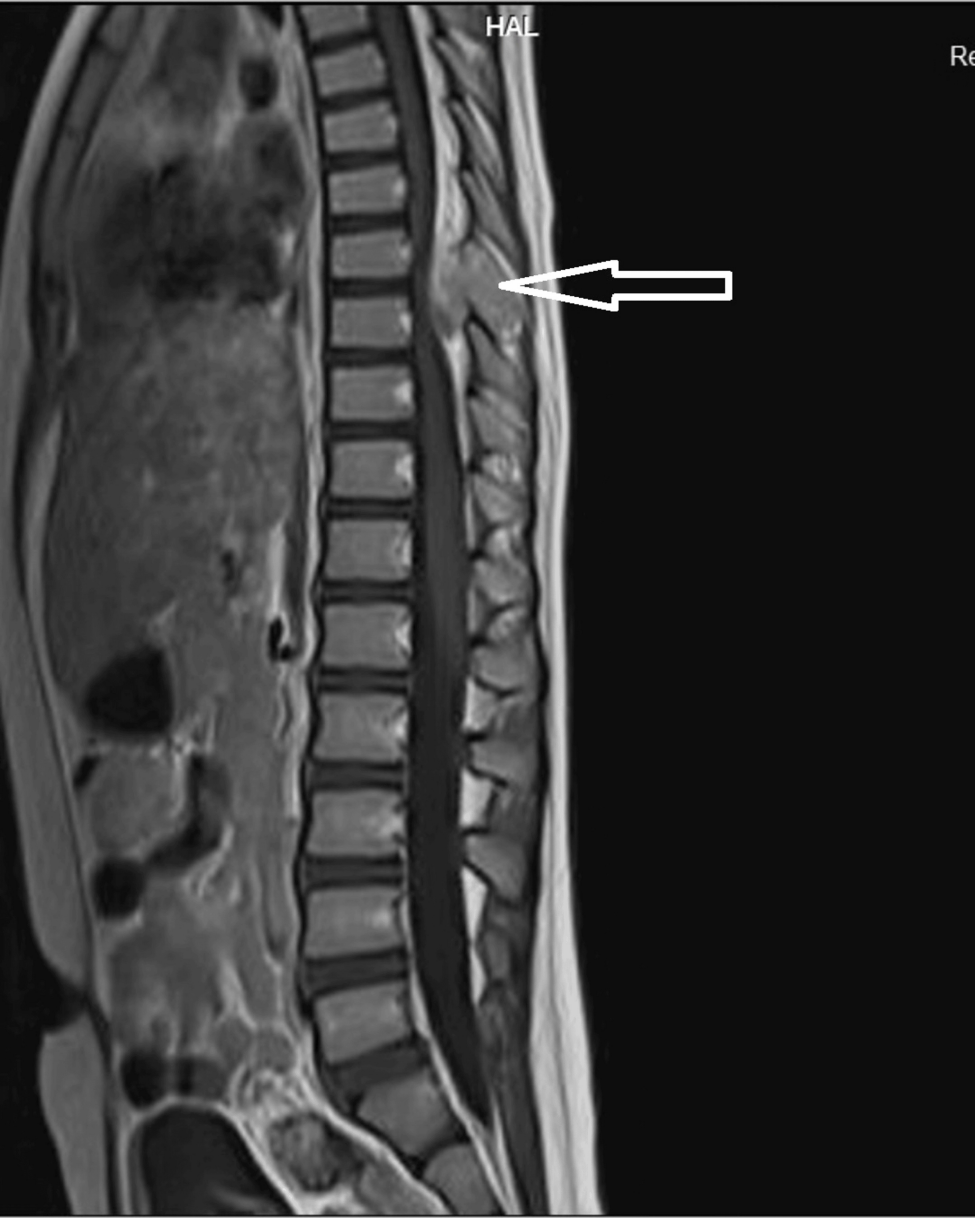
MRI image showing the aneurysmal bone cyst four years prior to admission

**Figure 2:**
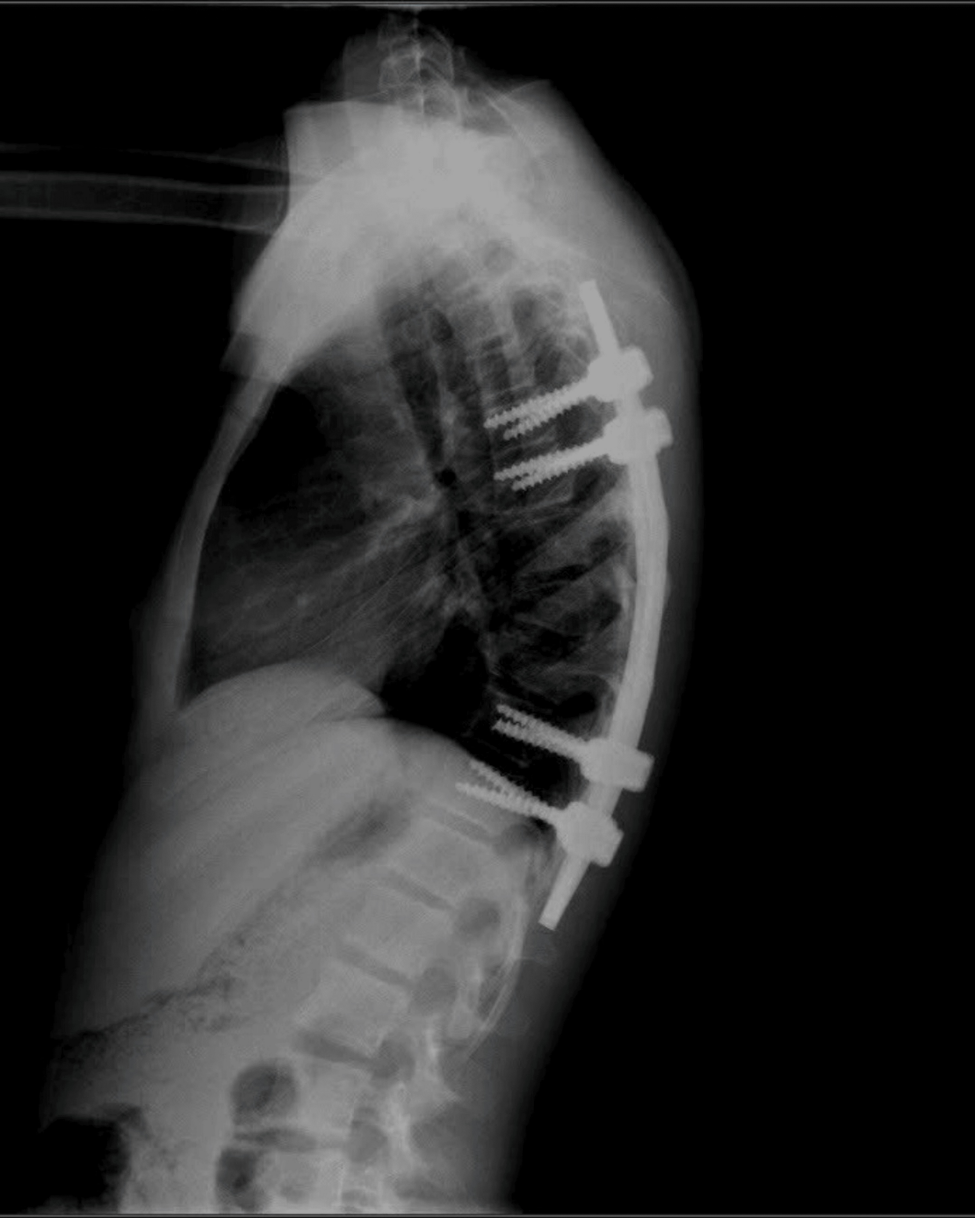
Imaging of the spinal frame 10 days after the second surgery

An 11-year-old girl was hospitalized due to daytime urinary incontinence, urgency, and nocturia persisting for four years. At the age of seven years, the patient was diagnosed with an ABC at the T8 vertebra, which was compressing the spinal cord after she experienced a sudden onset of lower limb weakness and urinary dysfunction. Magnetic resonance imaging (MRI) showed a cystic tumor structure (Figure 1). She underwent surgery to remove the cyst, followed by curettage and grafting of the damaged T8 vertebra. The spine was then stabilized with screws at T7 and T9. After surgery, she received physical therapy to rehabilitate her lower limbs. She was able to walk after nine months of intervention, albeit with a limp and weaker right leg compared to the left. For her urinary symptoms, she was diagnosed with an OAB based on urodynamic testing and was prescribed anticholinergic medication combined with CIC. However, the patient and her family could not adhere to the treatment due to lacking information about her condition and insufficient guidance on how to perform CIC properly. Four years post-surgery, the patient developed severe kyphosis, though she had no pain or other accompanying symptoms. A spinal MRI showed a collapse of the T8 vertebra, and she was scheduled for a second surgery to remove the two screws and replace them with a spinal frame spanning ten vertebrae from T3 to T12 (Figure 2). Ten days post-surgery, her spinal condition stabilized.

The clinical examination revealed that the child was fully conscious and had age-appropriate cognitive function; however, there was lower limb weakness, with muscle strength of 4/5 in the right leg and 5/5 in the left leg. The child could walk independently with a limp due to muscle strength discrepancy between the legs. The Barthel Index score was 100/100, indicating near-normal independence in daily activities. The Berg Balance Scale score was 51/56, indicating a low risk of falls. The assistance of an AFO*** brace significantly improved the child’s balance when walking. Therefore, this report will not address mobility and movement issues further.

**Figure 3:**
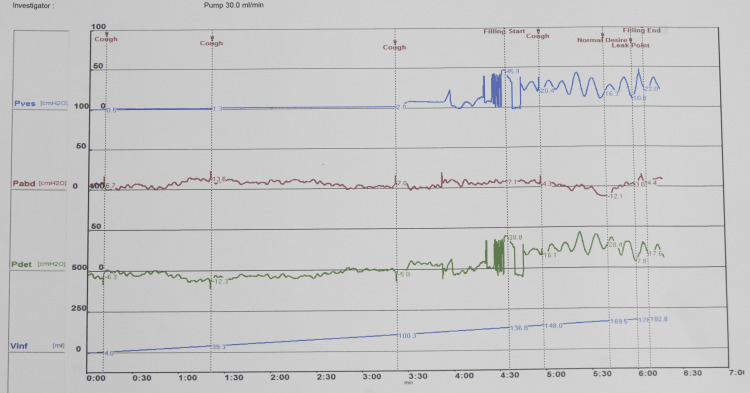
Urodynamic results before treatment

The child was frequently bothered by daytime urinary incontinence, urgency, and nocturia and always needed diapers during school hours. The Pediatric Quality of Life (PedsQL 4.0) score was 53. The OAB-SS score of 14 at admission reflected the severity of the symptoms. Abdominal ultrasound showed normal urinary tract structure without hydronephrosis. Urinalysis results did not indicate any urinary tract infection. Urodynamic testing revealed that the maximum detrusor pressure was 43.6 cmH_2_O when bladder capacity reached only 136.8 mL. The maximum bladder capacity was 156 mL. The first desire to void (FDV) occurred when the bladder capacity was 136 mL, with leak point (LP) occurring when the bladder volume reached 170 mL and the post-void residual volume was 145 mL (Figure 3). Based on the clinical symptoms and urodynamic findings, the child was diagnosed with an OAB. The treatment goals for the child were (a) to reduce the OAB-SS score to the average level, (b) to increase the maximum bladder capacity, (c) to decrease detrusor overactivity, (d) to reduce post-void residual volume, and (e) to improve the quality of life.

**Figure 4:**
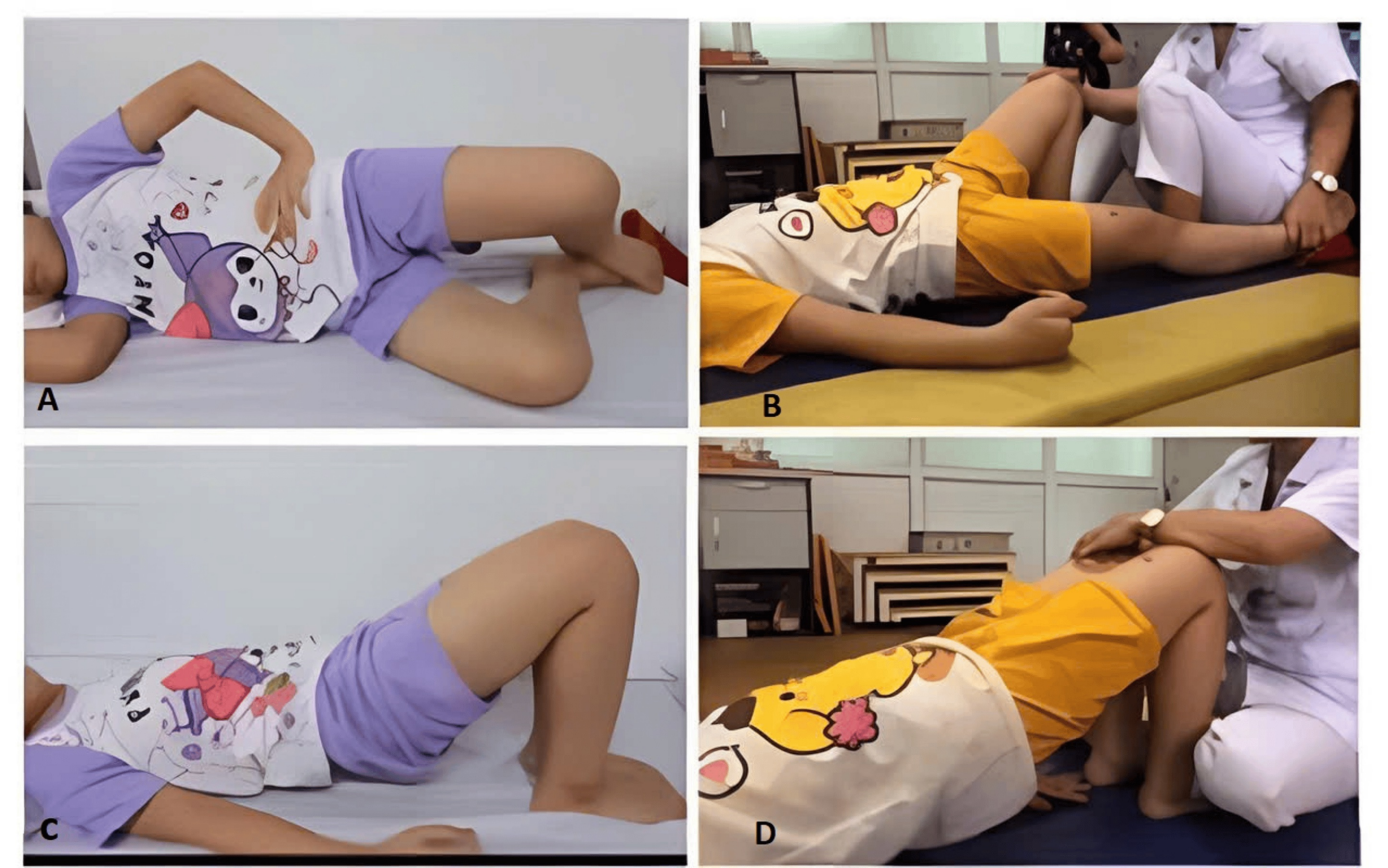
(A) Exercises for stretching and relaxing pelvic floor muscles. (B, C, and D). Exercises for strengthening glutes, inner thigh, and pelvic floor muscles.

Treatment indications for the child included four times of CIC per day, 5 mg of oxybutynin orally per day divided into two doses, and urotherapy. In standard urotherapy, the child was educated about the structure and function of the bladder using educational videos in Vietnamese on YouTube. Advice on drinking sufficient water throughout the day and maintaining an appropriate voiding frequency was given. The child received a bladder diary to track fluid intake and output and was encouraged to fill it out accurately. In specific therapy, the physician and therapist guided and assisted the child in timed voiding, bladder training, pelvic floor muscle training (Figure 4), central inhibition training, double voiding, and tibial nerve stimulation using transcutaneous electrical nerve stimulation (TENS). For CIC, the child and caregivers were instructed on how to use the CIC kit. In the first two weeks, the caregivers assisted the child with catheterization. After that, the child performed CIC independently, with the caregiver available for support if necessary.

After four weeks of treatment, most goals were achieved. Clinical symptoms, such as urinary frequency, significantly reduced, with no nocturia or only one instance per night. Urinary incontinence and urgency disappeared. The OAB-SS score dropped to 5, indicating that the clinical symptoms were mild, with no incontinence, nocturia, and fewer urgency episodes (Table 1).

**Table 1:** Overactive Bladder Symptom Score before and after treatment

	Frequency	Score	Before treatment	After treatment
How many times do you typically urinate from waking in the morning until sleeping at night?	≤7	0		0
	8-14	1	1	
	≥15	2		
How many times do you typically wake up to urinate from sleeping at night until waking in the morning?	0	0		
	1	1		1
	2	2		
	≥ 3	3	3	
How often do you have a sudden desire to urinate, which is difficult to defer?	Not at all	0		
	Less than once a week	1		
	Once a week or more	2		
	About once a day	3		
	2-4 times a day	4		4
	5 times a day or more	5	5	
How often do you leak urine because you cannot defer the sudden desire to urinate?	Not at all	0		0
	Less than once a week	1		
	Once a week or more	2		
	About once a day	3		
	2-4 times a day	4		
	5 times a day or more	5	5	
Total score			14	5

**Figure 5:**
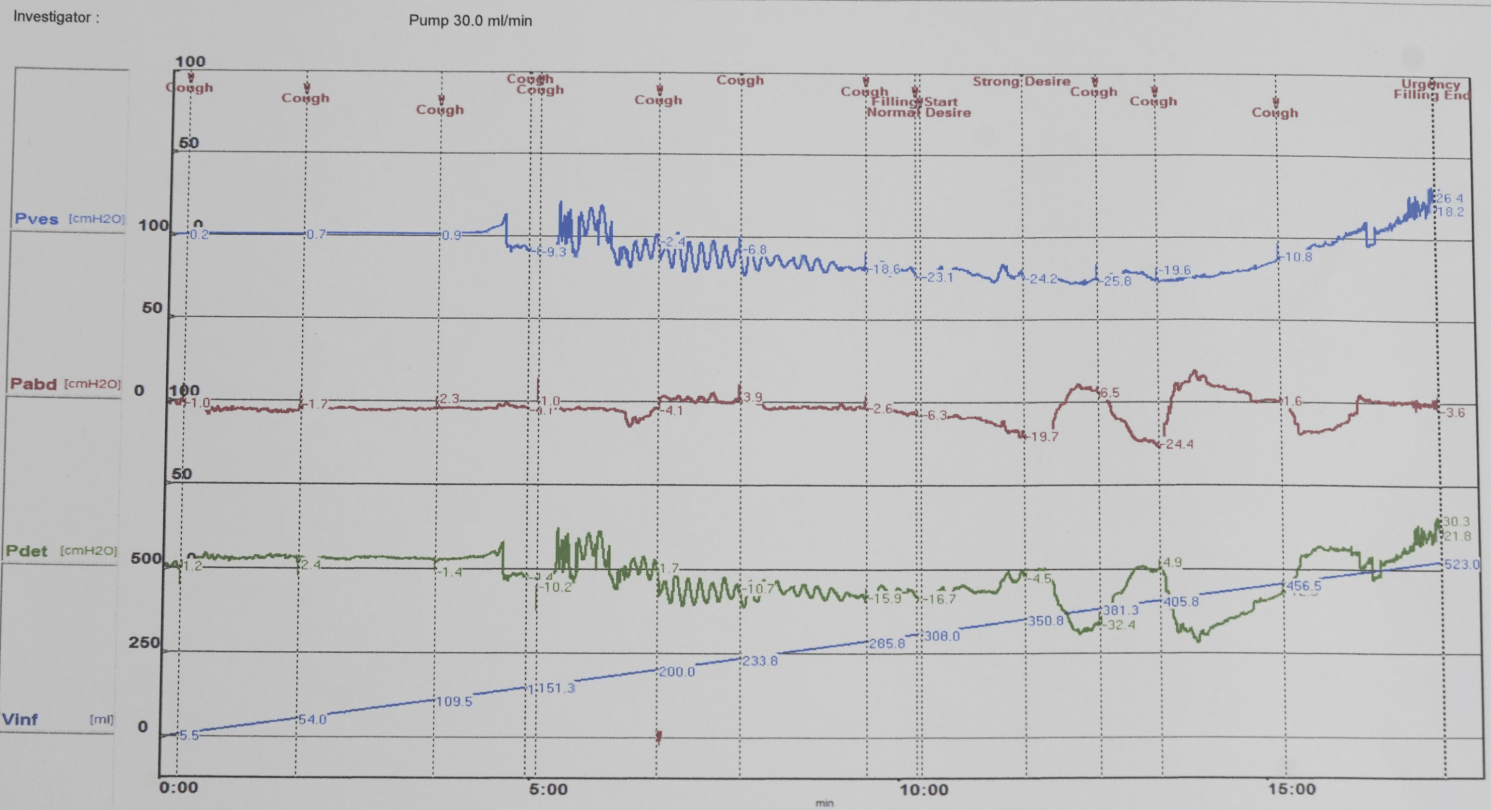
Urodynamic after six weeks of treatment

After six weeks of treatment, urodynamic results showed an increase in maximum bladder capacity (523 mL), with no more detrusor overactivity (maximum detrusor pressure was 30.3 cmH_2_O). The bladder sensation returned to normal, with the FDV occurring at 308 ml of bladder capacity, and there was no leak point during the procedure (Figure 5). The patient's quality of life improved, as shown by the reduction in the PedsQL score to 32. However, the post-void residual volume remained high (182 mL), which can lead to vesicoureteral reflux and urinary tract infections. Therefore, our goal for the next treatment phase was to reduce these consequences by continuing therapy and maintaining an appropriate frequency of CIC placement.

## Discussion

According to recent guidelines, the combination of medication, CIC, and urotherapy is the most optimal treatment for patients diagnosed with OAB [1]. This report presents the effectiveness of rehabilitation intervention including urotherapy, CIC, and medication in improving clinical symptoms and urodynamic outcomes for children with urinary disorders following spinal ABC surgery in Vietnam.

Primary ABCs account for approximately 1% of primary bone tumors, while spinal ABCs account for 15-20% of tumors in the spine [7]. Spinal ABCs are benign tumors situated near the spinal cord and nerve roots. They exhibit local progression and bone destruction, leading to neurological symptoms due to spinal cord compression and posing a risk of instability and bone deformity after surgery [7]. The traditional surgical treatment method involves curettage and bone grafting using instrumentation, with or without joint stabilization [8-10]. Symptoms of ABC include back pain, weakness or paralysis of the lower or all four limbs, neck pain, chest pain, sensory disturbances, and urinary or bowel dysfunction [11,12]. Among these symptoms, neurogenic bladder has been observed in 3 out of 17 patients with OABs of the spine in a patient series study by Flyer et al. and in 4 out of 10 patients with OABs of the sacrum in a case series study by Brastianos et al. [2,12]. The diagnosis of neurogenic bladder in children is based on urinary symptoms such as daytime urinary incontinence, leaked urine, urge incontinence, nocturnal enuresis, and increased detrusor pressure observed in urodynamic results. This condition can lead to complications such as urinary tract infection, vesicoureteral reflux, pyelonephritis, kidney failure, and reduced quality of life for both the child and their caregivers [1].

Anticholinergic medications are the first-line treatment for neurogenic detrusor overactivity. These drugs reduce detrusor muscle spasms by relaxing the muscle, consequently increasing bladder capacity. Anticholinergic treatment for OAB symptoms aims to alleviate discomfort from urgency and reduce urinary frequency and infrequent incontinence [4]. Oxybutynin is approved for use in children at 0.1-0.4 mg/kg/day, divided into two to three doses [1]. Its side effects include dry mouth, skin flushing, constipation, urinary retention, increased post-void residual volume, blurred vision, and cognitive disturbances at higher doses. In our patient, mild constipation was noted but managed effectively through dietary adjustments and short-term oral laxatives.

CIC is a standard treatment for neurogenic bladder. However, it is quite complex and requires multiple steps. Most adults can perform CIC independently, but children rely on their caregivers. Our patient had a prior diagnosis of OAB, but CIC was discontinued due to challenges in maintaining the frequent CIC regimen, difficulties in locating the catheterization site, and significant discomfort for the child, with minimal support from health professionals. CIC involves numerous challenges, including technical difficulties, the cost of catheters, frequent repetition throughout the day, and emotional factors [13]. There is limited knowledge of barriers to CIC adherence and caregiver experiences. Some studies have identified critical factors impacting CIC adherence, such as a lack of support from family and healthcare teams, insufficient time to adapt to the new routine, and starting CIC at an older age [13]. One study conducted among children aged 8-15 introduced the concept of “shared-CIC,” referring to the transition from caregiver-CIC to self-CIC. This study found that shared-CIC was most common in children aged eight to nine years, and self-CIC rates increased with age. Important predictors for increased self-CIC included less severe spinal lesions, higher CIC mastery, and lower CIC adherence [14]. Higher CIC adherence was noted when caregivers performed catheterization, when catheters were self-purchased, and when fewer catheterization challenges were encountered. However, adherence tended to drop as children began self-catheterizing. Therefore, healthcare providers should pay special attention during this transition phase. Some patients with spinal cord conditions experience significant pain during CIC, highlighting the need for reassessment and re-education in catheterization techniques to improve adherence [15].

Our observations in this study put forward some recommendations for urotherapy in children. ICCS guidelines describe urotherapy as a non-surgical, non-pharmacological (rehabilitative) intervention for lower urinary tract dysfunction in children and adolescents. Urotherapy specifically addresses symptoms such as daytime urinary incontinence, nocturnal enuresis, and constipation. The goal is to normalize urinary and bowel function and prevent dysfunction through consistent and ongoing practice. Urotherapy focuses on cognitive-behavioral therapy, including standard and specific urotherapy approaches. Standard urotherapy involves providing information, guidance, lifestyle advice, counseling, and symptom monitoring. Specific urotherapy is individualized based on each patient’s conditions and may include alarm therapy, biofeedback training, pelvic floor muscle exercises, neuromodulation, and other interventions. Our patient did not experience nocturnal enuresis, and thus alarm therapy was unnecessary. Although biofeedback has proven effective in treating urinary dysfunction, equipment was unavailable at our facility. Therefore, we applied pelvic floor exercises, tibial nerve stimulation using TENS, and bladder training exercises (timed voiding, double voiding, and deferment exercises) for this patient. Urotherapy aims to comprehensively address physical, psychosocial, behavioral, and quality-of-life aspects of patients. Thus, the ICCS recommends urotherapy as a first-line intervention for all lower urinary tract disorders [3].

## Conclusions

After the diagnosis of OAB following spinal ABC surgery, our patient was treated conservatively with a combination of rehabilitation interventions, including urotherapy, CIC placement, and anticholinergic medication. This combination aligns with recent recommendations. The recorded outcomes include a reduction in symptoms such as urinary incontinence, urgency, nocturia, decreased detrusor pressure, increased maximum bladder capacity, and an improvement in the child's quality of life.
